# Targeted gene silencing in human embryonic stem cells using cell-penetrating peptide PepFect 14

**DOI:** 10.1186/s13287-019-1144-x

**Published:** 2019-01-24

**Authors:** Egle-Helene Ervin, Martin Pook, Indrek Teino, Valmar Kasuk, Annika Trei, Margus Pooga, Toivo Maimets

**Affiliations:** 1 Department of Cell Biology, Institute of Molecular and Cell Biology, University of Tartu, Riia 23, 51010 Tartu, Estonia; 2 Laboratory of Molecular Biotechnology, Institute of Technology, University of Tartu, Nooruse 1, 50411 Tartu, Estonia

**Keywords:** Human embryonic stem cells, siRNA transfection, Cell-penetrating peptide, PepFect 14, OCT4, B2M

## Abstract

**Background:**

Human embryonic stem (hES) cells serve as an invaluable tool for research and future medicine, but their transfection often leads to unwanted side effects as the method itself may induce differentiation. On the other hand, RNA interference (RNAi)-based targeted gene silencing is a quick, cost-effective, and easy-to-perform method to address questions regarding the function of genes, especially when hypomorphic knockdowns are needed. Therefore, effective transfection method with minimal side effects is essential for applying RNAi to hES cells. Here, we report a highly promising approach for targeted gene silencing in hES cells with siRNA complexed with cell-penetrating peptide PepFect 14 (PF14). This strategy provides researchers with efficient tool for unraveling the functions of genes or addressing the differentiation of pluripotent stem cells.

**Methods:**

We present a method for delivery of siRNA into hES cells with cell-penetrating peptide PF14. Accordingly, hES cells were transfected in ROCK inhibitor containing medium for 24 h right after EDTA passaging as small cell clumps. Fluorescently labeled siRNA and siRNAs targeting OCT4 or beta-2-microglobulin (B2M) mRNA sequences were used to evaluate the efficiency of transfection and silencing. Analyses were performed at various time points by flow cytometry, RT-qPCR, and immunofluorescence microscopy.

**Results:**

Effective downregulation of OCT4 in 70% of treated hES cells at protein level was achieved, along with 90% reduction at mRNA level in bulk population of cells. The applicability of this low-cost and easy-to-perform method was confirmed by inducing silencing of another target not associated with hES cell pluripotency (B2M). Furthermore, we discovered that downregulation of OCT4 induces neuroectodermal differentiation accompanied by reduced expression of B2M during early stage of this lineage.

**Conclusions:**

The results demonstrate PF14 as a promising tool for studying gene function and regulatory networks in hES cells by using RNAi.

**Electronic supplementary material:**

The online version of this article (10.1186/s13287-019-1144-x) contains supplementary material, which is available to authorized users.

## Background

Human embryonic stem (hES) cells are pluripotent cells isolated from the inner cell mass of blastocyst stage embryo. These self-renewing cells are capable of differentiation into all cell types derived from three primary germ layers (ectoderm, endoderm, and mesoderm) [1]. Due to their proliferative and developmental capacity, hES cells hold great potential in cellular therapy, disease modeling, drug discovery, and for studying human early development. These clinical and research applications require quick, efficient, and robust methods for studying gene function by knocking down its expression in hES cells.

Self-renewal and differentiation of hES cells is under control of multiple genes and signaling pathways. For example, expression of transcription factors *OCT 4* [2], *SOX2*, and *NANOG* [3–5] as well as activation of FGF [6], PI3K/AKT, SMAD [7], and WNT [8] pathways regulate pluripotency and lineage commitment. To shed light on specific mechanisms governing differentiation and regulating hES cell self-renewal, additional studies are required. RNA interference (RNAi) technology is a powerful tool for assessing a gene’s function and essentiality in different regulatory networks, and it allows creation of hypomorphic knockdowns [9]. RNAi is a mechanism for post-transcriptional gene expression silencing where short double-stranded RNA initiates degradation of complementary mRNA [10]. One group of such functional RNAs are short interfering RNAs (siRNAs) which induce degradation of fully complementary mRNA with no mismatches [11]. Therefore, siRNA is considered as a precise and highly effective tool for regulating expression of a particular gene and has been successfully applied to silence various genes in different mammalian cell types [11, 12]. However, the highly anionic nature of siRNAs excludes direct crossing of the cell membrane posing transfection-related obstacles [11]. Delivery has actually been the main reason of limited success of harnessing RNAi in embryonic stem cell biology as hES cells are difficult to transfect with exogenous DNA or RNA [13]. The desired method should provide high transfection efficiency, low or no cytotoxicity, reproducibility, and be easy to use without interfering with normal physiology of hESC. The common non-viral transfection methods utilized in mammalian cell culture could be divided into two groups: cationic lipid or polymer-based delivery [14].

Lipofection is routinely used for transfection of human cells based on condensing anionic nucleic acids with cationic lipids to particles that are efficiently taken up by the cells. Although lipid-based carriers have shown promising results, double transfection and pre-plating of the cells 24 h prior experiment is time-consuming but are still required for achieving satisfactory efficiency and low cytotoxicity [3, 8, 15–18].

Peptide-mediated delivery relies on cell-penetrating peptides (CPPs), defined as short peptides able to cross biological barriers and facilitate cellular uptake of various cargo molecules. CPPs used for siRNA delivery contain multiple positively charged amino acid residues and form non-covalent complexes with negatively charged nucleic acids [19]. Formed nanoparticles are internalized by the cells mainly using endocytosis [20]. Different CPPs have been developed to date aiming efficient cellular delivery vectors that also liberate its payload from endosome that is crucial for cargo molecule functioning [19]. Recently, PepFects, a family of CPPs, were designed especially for nucleic acid delivery. Among these PepFect 14 (PF14), whose main advantages include low cytotoxicity, ability to form non-covalent nanocomplexes with oligonucleotides, high transfection efficiency, and independence from confluency [21–23]. PF14 has efficiently delivered splice-correcting oligonucleotides (SCOs), siRNA, and plasmid DNA (pDNA) in vitro and in vivo [21, 22].

Since cytotoxicity and low transfection efficiency are the main problems with other transfection reagents, we consider PF14 a promising tool for post-transcriptional gene silencing in hES cells. We propose an entirely novel approach as CPPs have been used to direct induced pluripotent stem cells (iPSCs) differentiation via protein transduction [24] and PF14 has been tested for pDNA delivery into mouse ES cells so far [22]. However, to our knowledge, CPPs have not been applied for siRNA delivery into hES cells. Altogether, combining hES cells, RNAi, and CPPs holds great promise for research and therapeutic applications.

## Methods

### Cell culture

hES cell line H9 (WA09) and H1 (WA01, both National Stem Cell Bank) was cultured on Matrigel (BD Biosciences)-coated 6-well tissue culture plates (Corning) in mTeSR1 medium (STEMCELL Technologies) which was changed daily. Cells were mechanically passaged every 3–4 days using micropipette tip for detaching and breaking the colonies into pieces followed by plating onto fresh Matrigel-coated plates. Prior to transfection cells were passaged with EDTA-PBS to achieve a suspension of small cell clumps (described below). Cells were cultured at 37 °C in 5% CO_2_ and in a humidified atmosphere.

### Passaging for transfection

hES cells were washed and incubated in 0.5 mM EDTA in phosphate-buffered saline (PBS). The plate was incubated on the warm surface (37 °C) for 8–10 min. Cell suspension was obtained by slow pipetting with micropipette for several times, and it was transferred to a 15-ml tube followed by centrifugation at 200 g for 5 min. Supernatant was removed, and cells were resuspended in fresh medium and counted by Countess II (ThermoFisher). Cell suspension with suitable concentration of cells was prepared, and 1 ml of suspension was added to each well. Three different plating densities were used, namely 3 × 10^5^ or 4.5 × 10^5^ or 6 × 10^5^ cells per well of 6-well plate.

### Formation of transfection complexes

PepFect 14 (PF14, sequence: stearyl-AGYLLGKLLOOLAAAALOOLL-NH_2_) was purchased from Pepscan and Alexa Fluor 568 conjugated siRNA (siRNA-Alexa Fluor 568) from Eurofins Genomics (sense: 5′-ACGCCAAAAACAUAAAGAAAG; antisense: 5′-UUCUUUAUGUUUUUGGCGUCU). Silencer® select pre-designed OCT4 siRNA (siOCT4, ID: s10872), B2M siRNA (siB2M, ID: s1854) and Negative control #1 siRNA (siCtrl) were obtained from Ambion (ThermoFisher).

PF14 1-mM aliquots were stored at − 20 °C. For transfection, aliquots were diluted to 100 μM with MQ water and used for maximum 3 independent experiments (i.e., maximum storage of 3 weeks at 4 °C).

On the day of experiment, 20-μM aliquots of siRNA in water were thawed and diluted in MQ water followed by addition of PF14 and mixing by pipetting. Complexes were formed in 200 μl (1/10th of final transfection volume) for 1 h at RT.

siRNA and PF14 concentrations were adjusted proportionally to achieve nanocomplexes at charge ratio (CR, also known as N/P) of 2:1 and molar ratio (MR) of 16:1 (peptide:siRNA). For controlled complex formation, a solution for 30 nM siRNA final concentration was prepared and the volume of solution added to the cells varied accordingly.

Transfection complexes of Lipofectamine Stem reagent (ThermoFisher) and siRNA (final concentration of 20 nM) were prepared according to manufacturer’s instructions.

### Transfection

hES cells were transferred onto Matrigel-coated plates as described above. Eight hundred microliters of mTeSR1 medium containing ROCK inhibitor Y-27632 (final concentration of 10 μM, Tocris Bioscience) was added to transfection solution and applied to freshly plated cells.

**Fig. 1:**
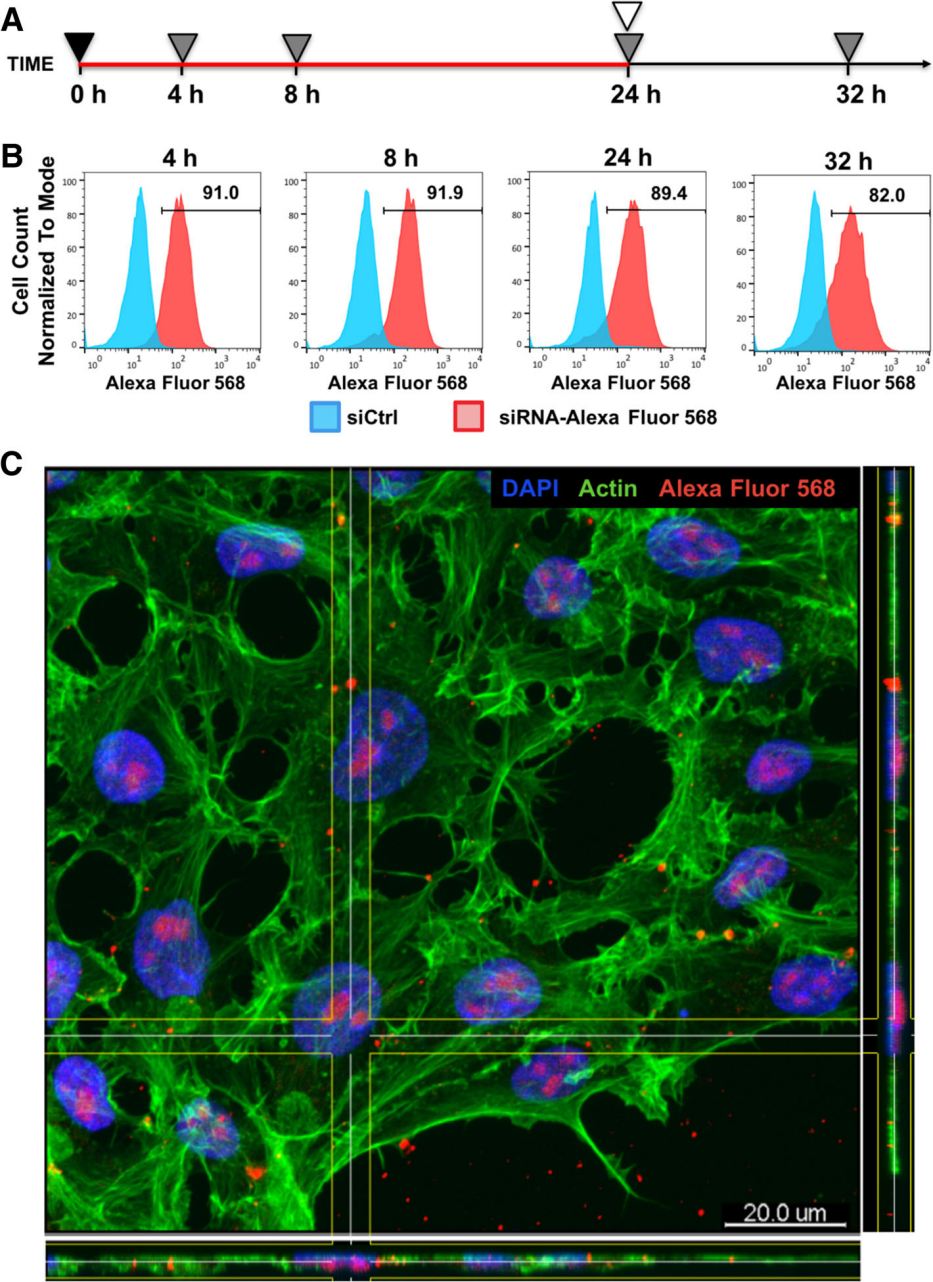
PF14 delivers fluorescently labeled siRNA into hES cells with high efficiency. **a** Scheme of experiment: plating of cells (4.5 × 10^5^ cells/well) and start of transfection at 0 h. Transfection complexes remained in the growth medium for 24 h. During that period, 3 independent flow cytometry analyses were performed (at 4 h, 8 h, and 24 h). After 24 h, the medium was changed for one without complexes and flow cytometric analysis was performed at 32 h. siRNA was used at concentration of 30 nM. Symbols: red line—transfection, black triangle—plating cells and adding transfection complexes, gray triangle—flow cytometric analysis, white triangle—medium exchange. **b** Flow cytometric analysis of siRNA-Alexa Fluor 568 in hES cells at various time points. Cells incubated with siCtrl/PF14 complexes were used as controls. Bars and numbers on histogram indicate the percentage of transfected cells in siRNA-Alexa Fluor 568-treated sample. **c** Immunofluorescence analysis of siRNA-Alexa Fluor 568 (red) localization in siRNA/PF14-treated cells after 24-h incubation. Cell nuclei were stained with DAPI (blue) and actin cytoskeleton with phalloidin (green). Scale bar is 20 μm

siRNA-Alexa Fluor 568 was transfected for 4–24 h, and transfection efficiency was estimated by flow cytometry shortly after the end of transfection or 8 h after the end of transfection (analysis at 32 h) as shown in Fig. 1a.

Transfection protocol was optimized with complexes containing siOCT4 applied for 24 h. Culture medium was exchanged for fresh medium without complexes every 24 h, unless the second transfection was performed at 24 h, 32 h, or 48 h time points. Cells from all setups were analyzed by flow cytometry at 72 h.

**Fig. 2:**
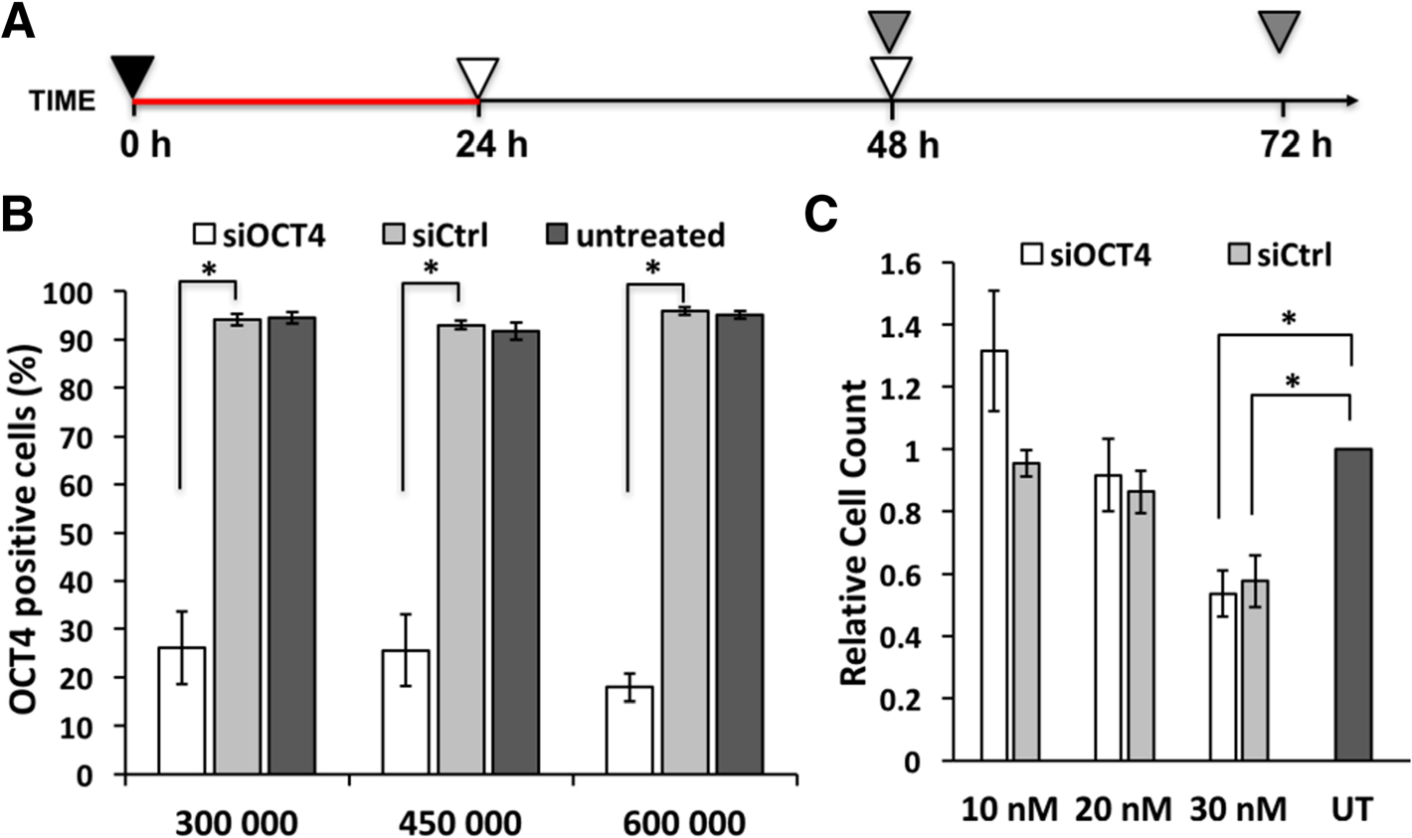
Cell viability and silencing efficiency depend on transfection conditions. **a** Scheme of the optimal gene silencing experiment: plating of cells (4.5 × 10^5^ cells/well) and start of transfection at 0 h. Transfection complexes remained in the growth medium for 24 h until the medium exchange that was repeated at 48 h. At 48 h and 72 h time points, OCT4 level was analyzed by flow cytometry. Symbols: red line—transfection, black triangle—plating cells and adding transfection complexes, gray triangle—flow cytometric analysis, white triangle—medium exchange. **b** Flow cytometric analysis of OCT4 expression at 72 h in untreated cells or cells treated with 30 nM siOCT4 or siCtrl presented as percentage of OCT4-positive cells. X-axis represents the number of the cells plated into a well of 6-well plate. **c** Cell count normalized to untreated sample at 72 h time point while using optimal protocol. Abbreviations: UT, untreated. Statistical significance with *P* values less than 0.05 are labeled as * (mean ± SEM, *N* = 3)

siOCT4 or siB2M was transfected for 24 h, and medium was changed as shown in Fig. 2a. Cells were analyzed at 48 h or 72 h.

In all experiments, cells incubated with siCtrl/PF14 complexes or without complexes (untreated) were used as controls. All time points refer to the time from the beginning of transfection.

### Flow cytometric analysis

Transfection efficiency was analyzed using siRNA-Alexa Fluor 568-transfected cells detached with 0.05% trypsin (Corning) for 3 min. Single cell suspension was diluted in PBS with 10% fetal bovine serum (FBS, Corning) and centrifuged at 300 g for 5 min. Cell pellet was resuspended in PBS containing 1% BSA (bovine serum albumin, Capricorn Scientific), 2 mM EDTA, and DAPI (0.5 μg/ml, Sigma-Aldrich); stored on ice; and analyzed by flow cytometry. Live and dead cells were distinguished by DAPI staining, and siRNA-Alexa Fluor 568 transfected and not transfected cells were discriminated based on siCtrl sample.

For analysis of OCT4, NANOG, SOX2, and B2M proteins’ levels, cells were harvested with 0.5 mM EDTA for 10 min. Single-cell suspension was diluted in PBS containing 1% BSA and 2 mM EDTA and cells counted by Countess II. 3 × 10^5^ cells were fixed in 1 ml of 1.6% paraformaldehyde (PFA, Sigma-Aldrich) in PBS for 10 min at RT, washed twice with PBS and permeabilization buffer (eBioscience), and resuspended in 50μl of permeabilization buffer containing fluorescent label conjugated antibodies or appropriate isotype controls (detailed information in Table 1). After 45 min of incubation at RT, cells were washed twice with permeabilization buffer and counterstained with DAPI (0.5 μg/ml). Samples were filtered and analyzed with FACSAria or FACSMelody (BD Biosciences), and data processed using FACSDiva and FlowJo software (BD Biosciences). Compensation was set with single-stained probes with CompBeads (BD Biosciences) and verified with single-stained cells. Negative population was set according to FMO and isotype controls. For OCT4, siRNA-induced silencing resulted in discrete populations and gating was performed based on this discrimination. For statistical analysis, geometric Mean fluorescence intensity (gMFI) of whole or gated population as well as proportion of cells in positive gate was used.

**Table 1:** Antibodies used for flow cytometric analysis and immunofluorescence microscopy

	Name	Dilution	Host and clonality	Supplier
Target protein				
OCT4 (Alexa 647)	653710 (3A2A20)	1:100	Mouse monoclonal IgG2b	BioLegend
NANOG (PE)	560483 (N31-355)	1:5	Mouse monoclonal IgG1	BD Biosciences
SOX2 (PerCp-Cy5.5)	561506 (030-678)	1:20	Mouse monoclonal IgG1	BD Biosciences
B2M (Alexa 488)	FAB8248G (883028)	1:20	Mouse monoclonal IgG1	R&D Systems
Isotype controls				
Alexa 647 conjugate	400330	1:100	Mouse IgG2b	BioLegend
PE conjugate	554680	1:80	Mouse IgG1	BD Biosciences
PerCp-Cy5.5 conjugate	552834	1:5	Mouse IgG1	BD Biosciences
Alexa 488 conjugate	557702	1:25	Mouse IgG1	BD Biosciences

Fluorescent label conjugated to antibody is indicated in the brackets of target protein column. Abbreviations: *PE* phycoerythrin, *PerCP* peridinin chlorophyll protein complex

### Immunofluorescence microscopy

To analyze siRNA-Alexa Fluor 568 localization, cells grown on 12-mm round cover glasses (Marienfeld) coated with Matrigel were transfected for 24 h. Cells were fixed with 4% PFA in PBS for 10 min at RT, washed twice with PBS, and blocked with 1% BSA and 0.1% saponin solution in PBS for 20 min at RT. Actin cytoskeleton was visualized with Alexa Fluor 488-phalloidin (165 nM, ThermoFisher), and cell nuclei with DAPI (0.5 μg/ml) incubation for 15 min at RT.

For analysis of OCT4 and B2M, cells were fixed at 72 h with 4% PFA in PBS for 10 min, washed twice, and blocked with 4% normal goat serum (NGS, Capricorn) and 0.1% saponin solution in PBS for 60 min at RT. Cells were incubated with antibodies (detailed information in Table 1) diluted in 0.1% saponin containing PBS for 60 min at RT. Cell nuclei were stained with DAPI as shown above.

Slides were mounted with Fluorescence Mounting Medium (Dako) and analyzed using confocal microscope LSM710 (Zeiss), and images were processed with Imaris software (Bitplane AG).

### RNA isolation, cDNA synthesis, and quantitative PCR

Cells were lysed in FARB buffer (Favorgen) containing 2-mercaptoethanol (1%, Sigma-Aldrich). RNA was extracted using FavorPrep Blood/Cultured cell Total RNA Mini Kit (Favorgen) according to manufacturer’s instructions, and RNA concentration was measured using spectrophotometer (NanoDrop 1000, ThermoFisher). One microgram of RNA was used for further analysis. Genomic DNA was digested by RNase free DNase I (ThermoFisher). cDNA was synthesized using RevertAid First Strand cDNA Synthesis Kit (ThermoFisher) with random hexamer primers in accordance with manufacturer’s instructions. Quantitative PCR (qPCR) analysis was carried out using Maxima SYBR Green qPCR Master mix (ThermoFisher) and specific primers (listed in Table 2). The amplification consisted of 95 °C for 10 min followed by 40 cycles of 95 °C for 10 s and 59 °C for 1 min analyzed by LightCycler 480 II Real-Time PCR System (Roche). The results were analyzed using LightCycler 480 software version 1.5 (Roche). Recorded threshold cycle (Ct) values from triplicate were averaged and normalized against endogenous reference gene *TATA-binding protein* (*TBP*). Relative mRNA level was calculated using ΔΔCt method [25].

**Table 2:** Primers used in polymerase chain reaction

Gene name	Primer sequence
B2M	Forward 5′-TGCTGTCTCCATGTTTGATGTATCT-3′
	Reverse 5′-TCTCTGCTCCCCACCTCTAAGT-3′
BRA	Forward 5′-ATGACAATTGGTCCAGCCTT-3′
	Reverse 5′-CGTTGCTCACAGACCACAG-3′
GATA4	Forward 5′-GCTCCTTCAGGCAGTGAGAG-3′
	Reverse 5′-CTGTGCCCGTAGTGAGATGA-3′
HAND1	Forward 5′-TGCCTGAGAAAGAGAACCAG-3′
	Reverse 5′-ATGGCAGGATGAACAAACAC-3′
NANOG	Forward 5′-CCTGTGATTTGTGGGCCTG-3′
	Reverse 5′-GACAGTCTCCGTGTGAGGCAT-3′
OTX2	Forward 5′-GCTGGCTATTTGGAATTTAAAGG-3′
	Reverse 5′-GGGTTTGGAGCAGTGGAAC-3′
PAX6	Forward 5′-AACAGACACAGCCCTCACAAAC-3′
	Reverse 5′-CGGGAACTTGAACTGGAACTGAC-3′
POU5F1 (OCT4)	Forward 5′-CTGGAGCAAAACCCGGAGG-3′
	Reverse 5′-CCTCAAAGCGGCAGATGGTC-3′
RPL13A	Forward 5′-CCTGGAGGAGAAGAGGAAAGAGA-3′
	Reverse 5′-TTGAGGACCTCTGTGTATTTGTCAA-3′
SOX1	Forward 5′-CAGCAGTGTCGCTCCAATTCA-3′
	Reverse 5′-GCCAAGCACCGAATTCACAG-3′
SOX2	Forward 5′-GTATCAGGAGTTGTCAAGGCAGAG-3′
	Reverse 5′-TCCTAGTCTTAAAGAGGCAGCAAAC-3′
SOX17	Forward 5′-GGCGCAGCAGAATCCAGA-3′
	Reverse 5′-CCACGACTTGCCCAGCAT-3′
TBP	Forward 5′-TGCACAGGAGCCAAGAGTGAA-3′
	Reverse 5′-CACATCACAGCTCCCCACCA-3′

### Induction of neural differentiation

STEMdiff™ Neural Induction Medium (STEMCELL Technologies) was used according to the manufacturer’s instructions.

### Statistical analysis

Values in all graphs are presented as mean ± SEM (standard error of the mean). Statistical tests were performed with three independent replicates using two-tailed unpaired Student’s *t* test. *P* values less than 0.05 were considered statistically significant.

## Results

### PepFect 14 mediates delivery of siRNA into hES cells with high efficiency

To assess whether PF14 [21] is able to transfer cargo into hES cells, fluorescently labeled siRNA (siRNA-Alexa Fluor 568) at 30 nM final concentration was used. siRNA without label and intracellular target in hES cells served as a negative control. Cells were subjected to flow cytometric analysis during transfection at 4 h, 8 h, 24 h, or 8 h post-transfection at 32 h time points (Fig. 1a). In order to minimize interfering extracellular membrane-bound signal, cells were detached from substrate with trypsin which removes membrane-associated siRNA complexes [26].

Transfection with PF14 complexes for 4 h is sufficient to transfer siRNA into majority of the hES cells as 91% of live cells appeared to be Alexa Fluor 568-positive (Fig. 1b). Furthermore, siRNA concentration in cells remained stable as long as siRNA and PF14 complexes were present in culture medium. After medium exchange, the proportion of Alexa Fluor 568-positive cells dropped slightly and remained at 82% level. To confirm that the detected signal originates from intracellular rather than membrane-associated siRNA, immunofluorescence analysis with confocal microscopy was performed. Alexa Fluor 568 signal from inside of the cells corroborated effective siRNA delivery into cell interior (Fig. 1c). Thus, PF14 transfers siRNA into hES cells within 4 h and ensures constant siRNA content as long as transfection complexes are present in cell medium.

### Transfection protocol optimization for efficient knockdown and high viability

Next, we analyzed the capability of PF14 to deliver functional (OCT4-targeting) siRNA into hES cells. Studies on lipofection have shown that transfection efficiency depends on many factors, including passaging method and the number of plated cells but most importantly on the number and frequency of transfections. To find out the optimal conditions for gene silencing, we used passaging as small cell clumps (2–5 cells), plated 4.5 × 10^5^ cells/well, and tested whether single or double transfection must be performed using 30 nM siOCT4. In both scenarios, the first transfection was performed immediately after plating the cells and lasted for 24 h. The second transfection was carried out at 24 h (i.e., no time for cells to recover in medium without complexes), 32 h or 48 h time points (i.e., cells recovered in fresh medium for 8 or 24 h between transfections, respectively). Cells incubated with non-targeted siRNA/PF14 complexes (siCtrl) and without siRNA/PF14 complexes (untreated) were used as controls. All cells were subjected to flow cytometric analysis at 72 h to quantify knockdown. As a result, over 85% of the cells were detected as OCT4-negative in siOCT4-treated samples without remarkable differences between used strategies (Additional file 1). However, substantially lower number of cells survived two transfections compared to a single transfection or untreated samples. Therefore, we preferred a strategy with single transfection (Fig. 2a) as it is less time-consuming and as effective as double transfection.

Efficacy of transfection reagents is usually influenced by cell density, but PF14 has shown to be insensitive to cell count [21]. To test, whether plating a particular number of hES cells is critical for successful transfection, 3 × 10^5^ and 6 × 10^5^ cells per well of 6-well plate were seeded in parallel with previously used 4.5 × 10^5^ cells. Although, 6 × 10^5^ cells/well yielded slightly more efficient knockdown, which may partly be achieved through a spontaneous differentiation in dense culture, various plating densities showed no markedly different efficiency (Fig. 2b). Thus, we chose to use lower cell densities to study the effect of siRNA and performed following experiments by plating 4.5 × 10^5^ cells/well.

During the optimization, we counted cells prior to the flow cytometric analysis and noted a trend of collecting less cells from siRNA/PF14-treated samples than from untreated samples. This indicates that siRNA (30 nM) and/or PF14 affect the cell viability (Fig. 2c) [27]. To further optimize protocol and increase survival rates, we performed dosage studies, where siRNA and PF14 concentration was lowered proportionally. As a result, we identified that 10 nM and 20 nM siRNA treatments do not significantly alter hES cell viability (Fig. 2c) and are preferred to use instead of 30-nM siRNA treatment.

### PepFect 14-mediated delivery of OCT4-specific siRNA effectively silences target in over 70% of hES cells

**Fig. 3:**
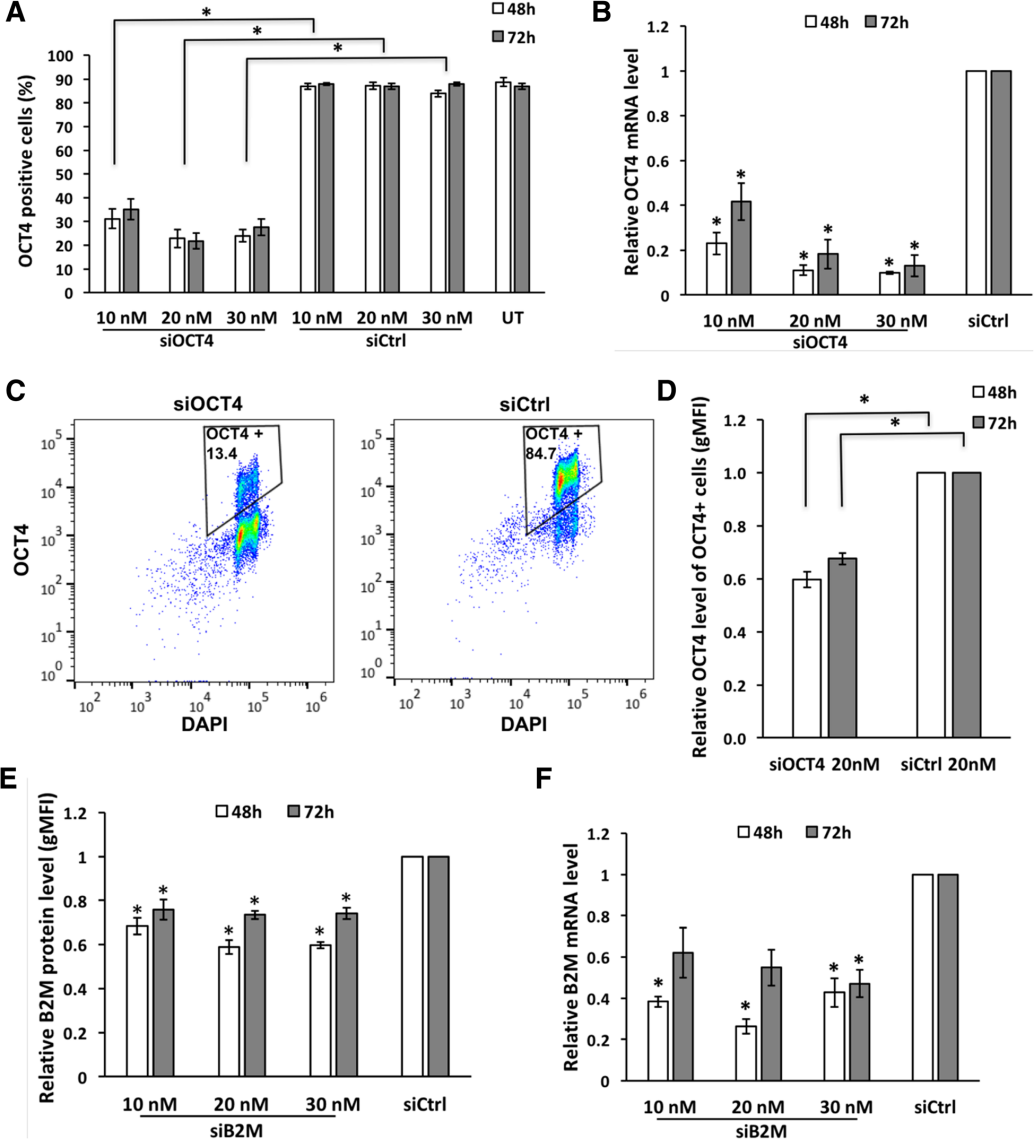
siOCT4 and siB2M silence targeted gene. **a** Flow cytometric analysis of OCT4 expression in cells treated with various concentrations of siOCT4 or siCtrl complexes with PF14, analyzed at 48 h and 72 h. **b** RT-qPCR analysis of *OCT4* mRNA level after silencing with siOCT4. **c** Representative dot blot of flow cytometric analysis illustrating lower proportion of OCT4-positive cells at 72 h in sample treated with 20 nM siOCT4. **d** Flow cytometric analysis of OCT4 protein level (estimated based on gMFI change) in OCT4-positive cells at 48 h and 72 h. **e** Flow cytometric analysis of B2M protein level (estimated based on gMFI change) after treatment with siB2M. **f** RT-qPCR analysis of *B2M* mRNA level after treatment with siB2M. All values on graphs (**b**, **e**, and **f**) are represented relative to siCtrl sample with identical concentration. Statistical significance with *P* values less than 0.05 are labeled as * (mean ± SEM, *N* = 3). Abbreviations: UT, untreated

Using optimized conditions and varying siRNA concentrations in parallel, we studied the extent of OCT4 silencing induced by respective siRNA. Flow cytometric analysis revealed that around 25% of cells were OCT4-positive in 20 nM and 30 nM siOCT4-treated samples compared to nearly 90% in untreated and siCtrl samples at both time points studied (namely 48 h and 72 h, Fig. 3a). Taking 10 nM siRNA concentration also into account, roughly 70% knockdown was achieved with every siRNA concentration used.

Our results showed that the silencing of expression was caused by general siRNA pathway that includes degradation of mRNA [28]. The latter was confirmed by RT-qPCR which indicated a decrease of OCT4 mRNA level up to 90% compared to siCtrl-treated cells (Fig. 3b). Although we admit that the mRNA and protein levels in the cell are not necessarily correlated, the discrepancy between efficiencies detected by flow cytometry and RT-qPCR can be explained by the fact that the results of RT-qPCR show average of the bulk population instead of single-cell resolution analysis by flow cytometry. The latter revealed a small population of OCT4-positive cells among the treated cells (Fig. 3c), which contributes to the result of mRNA level analysis. Moreover, we detected a drop in the mean (gMFI) of OCT4 protein level measured within OCT4-positive gated cell population, comparing siOCT4- and siCtrl-treated samples (Fig. 3d).

Regarding to mRNA levels, a clear dosage effect was evident supporting specific action of siRNA (Fig. 3b). At 48 h, the relative OCT4 mRNA levels in 10 nM, 20 nM, and 30 nM siOCT4-treated samples were 0.23, 0.11, and 0.10, respectively. Furthermore, a slight increase in OCT4 expression (relative mRNA level change from 0.23 to 0.42) was observed at 72 h while using 10 nM siRNA, which could be caused by the overgrowth of the cells that retained high OCT4 expression. To conclude, 20-nM siRNA concentration would be the optimal for downregulation of a target gene, if taking into account both the silencing efficiency and the cell viability.

We used optimal concentration of siOCT4 to compare the efficiency of PF14 to commercially available lipid-based transfection reagent Lipofectamine Stem (LF Stem), which is specifically developed for transfection of stem cells. To enable direct comparison, same strategy of passaging and transfection was used for both reagents; however, siRNA/LF Stem complexes were prepared according to the manufacturer’s protocol. As a result, a 70% reduction in OCT4 mRNA level was achieved upon transfection with LF Stem, while decrease of up to 90% was detected in cells treated with PF14 (Additional file 2). Thus, PF14 facilitates more efficient gene silencing than is achieved by LF Stem-mediated siRNA delivery.

### Single transfection induces moderate decrease in B2M mRNA and protein levels

Next, to further confirm PF14 applicability for knockdown in hES cells, we chose beta-2-microglobulin (*B2M*) as a target gene. *B2M*, as a component of major histocompatibility complex I, is a source of immunogenicity in rejection of hES cell-derived transplant. Thus, *B2M* knockout and knockdown studies have been performed earlier, which have confirmed that lowered levels of B2M do not suppress the self-renewal ability and pluripotency of hES cell [29–32].

We analyzed dose response to siB2M using concentrations of 10 nM, 20 nM, and 30 nM followed by analysis at 48 h and 72 h time points. Gene silencing at mRNA and protein level was achieved with all siRNA concentrations and at both time points used (Fig. 3e and f). However, 10 nM siB2M had the smallest effect with around 33% reduction of protein level at 48 h, compared to approximately 40% when using 20 nM and 30 nM siRNA. A small recovery in gene expression was observed at 72 h when the B2M silencing dropped to 25%. These findings were corroborated by RT-qPCR analysis, which showed increased mRNA level at 72 h compared to 48 h. In concordance with that, 20 nM siB2M lowered mRNA level by about 75% and 45% at 48 h and 72 h time points, respectively. More extensive suppression of mRNA compared to protein levels suggests effective and specific functioning of siRNA and indicates the stability of B2M protein. Thus, siB2M could induce modest and short-term silencing of its target.

Targeting of B2M was expected to not induce changes in the expression of OCT4. This would additionally confirm that the transfection method by itself does not influence the pluripotent status of hES cell. In concordance with that, no morphological changes indicative of differentiation were present in siB2M-treated samples (Additional file 3). Furthermore, siB2M had no significant impact on OCT4 expression (further evidence in next section, Fig. 5). Thus, we conclude that siB2M and PF14 as well as the knockdown of B2M do not influence expression of pluripotency factors in hES cell.

### PF14 works efficiently in both H9 and H1 hES cell lines

In order to determine whether transfection with PF14 is generally suitable for other hES cell lines, we transfected H1 cells with siOCT4, siB2M, and siCtrl (final concentrations of 20 nM). Analysis of mRNA levels at 48 h showed equally efficient knockdown of target genes in H1 as previously shown in H9 (Additional file 2). Accordingly, relative mRNA levels of OCT4 and B2M were 0.15 and 0.25 upon transfection with respective siRNA. Importantly, transfection with PF14 does not induce differentiation of H1 cells as no significant change in the expression of pluripotency factors was detected (Additional file 2). We conclude that PF14 is a highly efficient transfection reagent generally applicable for the studies involving hES cells.

### Knockdown of OCT4 induces differentiation of hES cells and upregulation of neuroectodermal markers

**Fig. 4:**
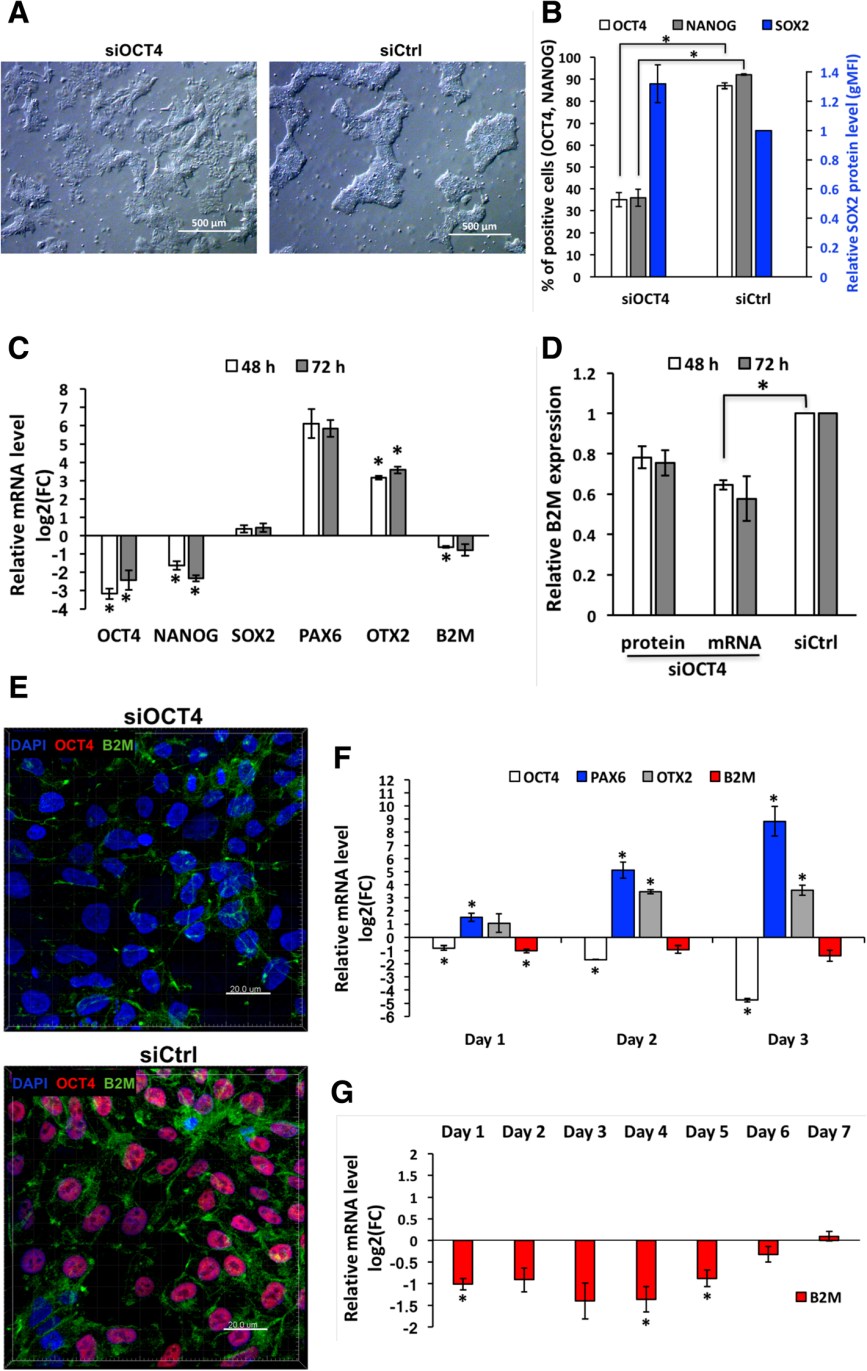
Expression of B2M is downregulated during early neuroectodermal differentiation induced by OCT4 silencing or neural induction medium. **a** Representative light micrographs illustrating changes in hES cell morphology at 72 h caused by treatment with 20 nM siOCT4. Scale bar is 500 μm. **b** Flow cytometric analysis of core pluripotency factor expression at protein level at 72 h in cells treated with 20 nM siOCT4, represented as percentage of cells positive for OCT4 and NANOG, and as a protein level (estimated according to gMFI change) normalized to siCtrl for SOX2. For gating strategy, see the “Methods” section. **c** RT-qPCR analysis of representative pluripotency and differentiation markers expression upon treatment with 20 nM siOCT4. mRNA level is presented as logarithm base 2 of the fold change in gene expression between the siCtrl and siOCT4 sample with identical concentration. **d** Flow cytometry and RT-qPCR analysis of B2M expression upon treatment with siOCT4. Values represented are relative to siCtrl sample with identical concentration (20 nM). **e** Representative fluorescence micrographs of OCT4 (red) and B2M (green) expression at 72 h in samples treated with 20 nM siOCT4 or siCtrl. Cell nuclei were stained with DAPI. Scale bar is 20 μm. **f**–**g** RT-qPCR analysis of representative pluripotency and differentiation markers and B2M expression during growth in neural induction medium. mRNA level is presented as a logarithm base 2 of the fold change in gene expression between cells grown in mTeSR1 and neural induction medium. Abbreviations: FC, fold change. Statistical significance with *P* values less than 0.05 are labeled as * (mean ± SEM, *N* = 3)

**Fig. 5:**
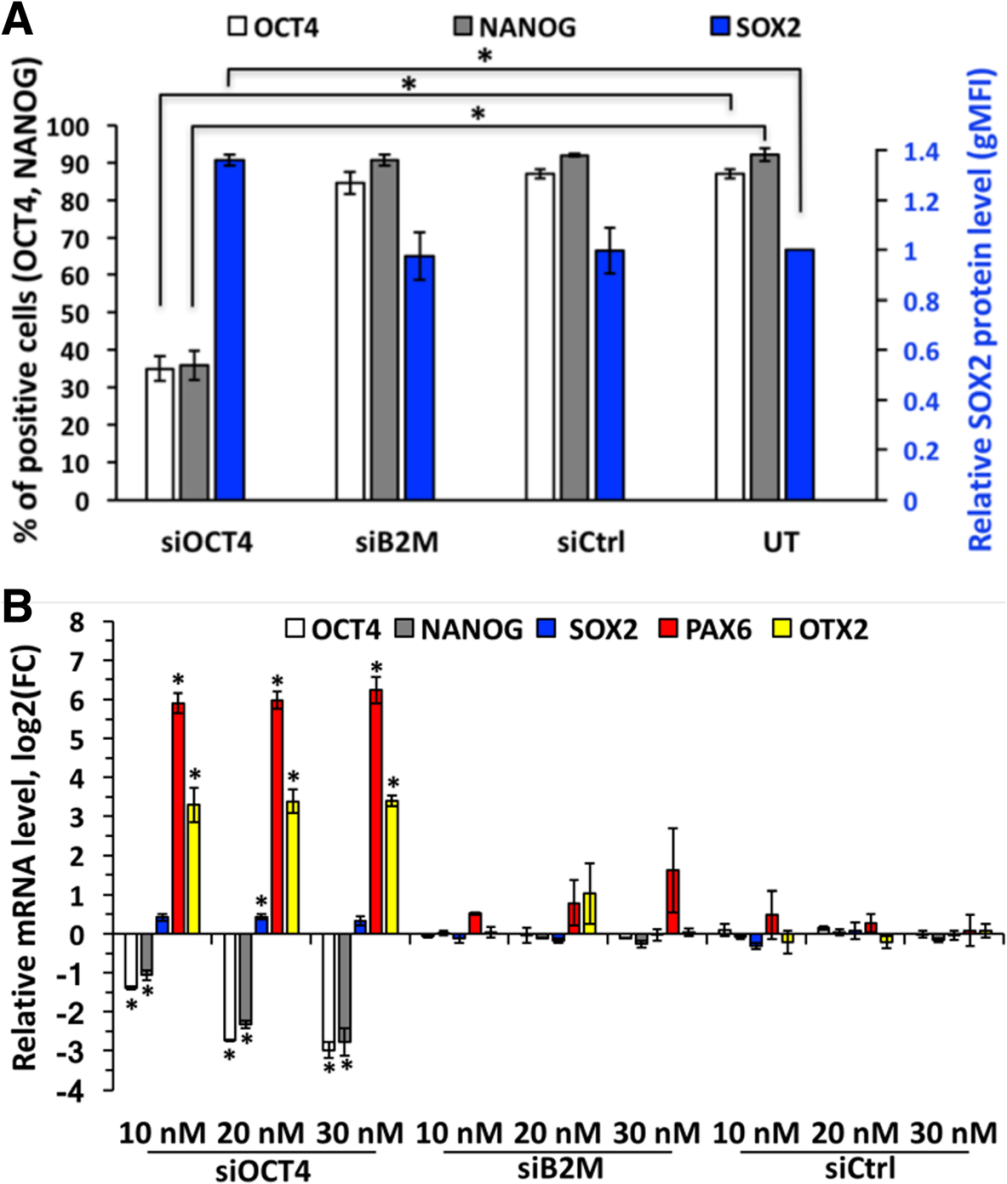
Treatment with siB2M or siCtrl does not influence expression of pluripotency factors in hES cells. **a** Flow cytometric analysis of core pluripotency factors expression at protein level at 72 h in cells treated with 20 nM siRNA, represented as percentage of positive cells for OCT4 and NANOG, and as a protein level (estimated according to gMFI change) relative to untreated sample for SOX2. For gating strategy, see the “Methods” section. **b** RT-qPCR analysis of representative pluripotency and differentiation markers’ expression upon treatment with siOCT4, siB2M, or siCtrl. mRNA level is presented as logarithm base 2 of the fold change in gene expression between untreated and siRNA treated sample. Statistical significance with *P* values less than 0.05 are labeled as * (mean ± SEM, *N* = 3). Abbreviations: UT, untreated

OCT4 is known as a factor essential for hES cell pluripotency and self-renewal [2, 8, 18, 33]. In accordance to that, first signs of differentiation after treatment with siOCT4 could be noticed by bright-field microscopy already within 24 h (Additional file 3). The changes in cell morphology were clearly evident at 48 h and 72 h time points, when siOCT4-treated cells failed to form dense colonies characteristic to hES cells (Fig. 4a). In addition, these cells had larger size compared to the cells treated with siCtrl or left untreated. To investigate, whether morphological changes were accompanied by changes in other pluripotency and self-renewal related factors, we estimated the protein and mRNA levels of NANOG and SOX2. In agreement with previous findings, expression of NANOG correlates with that of OCT4 [8, 33–35] and no statistically significant change in expression of SOX2 was caused by OCT4 silencing [36] (Fig. 4b). To further investigate the lineage specification following downregulation of OCT4, we analyzed expression of differentiation markers at mRNA level and detected remarkable increase in levels of neuroectodermal markers PAX6 and OTX2 (Fig. 4c). In contrast, siB2M and siCtrl samples did not induce significant changes in OCT4 and other pluripotency or differentiation related markers, evidently excluding any unspecific effects (Fig. 5). Altogether, these results indicate that downregulation of OCT4 in hES cells (at least H9 cell line) grown in chemically defined (mTeSR1) medium and on Matrigel-coated plates triggers differentiation of cells towards neuroectodermal lineage, which is in concordance with earlier studies [36]. However, to confirm the finding of lineage commitment, a longer follow-up study is required. Nevertheless, these results confirm usefulness of our method for studying the early embryonic differentiation and gene regulation networks and confirm that the method per se does not alter the characteristics of hES cell.

### Expression of B2M decreases during neuroectodermal differentiation induced by OCT4-specific siRNA

During all our experiments, the levels of both B2M and OCT4 were measured in parallel and surprisingly revealed an effect of siOCT4 treatment on B2M levels. Treatment with 20 nM siOCT4 knocked B2M down by 20% at protein level at 48 h and 72 h time points (Fig. 4d). Downregulation of B2M upon reduction of OCT4 levels was also confirmed by immunofluorescence microscopy, where cells with low or no OCT4 expression had clearly lower B2M levels (Fig. 4e). Moreover, 20 nM siOCT4 induced nearly a 2-fold decrease in B2M mRNA level, which also persisted at 72 h (Fig. 4d). The fact that downregulation of OCT4 induced stable decline in B2M mRNA level indicates a specific regulation mechanism during differentiation and excludes the possibility that the silencing of B2M is a result of siOCT4 off-target effect.

Previous studies have shown that the expression of B2M is upregulated during hES cell differentiation in embryoid body culture [37]. To clarify the discrepancy with our findings, we used neural induction medium to promote hES cell differentiation towards neural lineages. We detected a decrease in the levels of OCT4 and an increase in the levels of PAX6 and OTX2 at day 3, which is in agreement with neuroectodemal differentiation triggered by OCT4 silencing (Fig. 4f). Furthermore, we discovered nearly a 3-fold decline of B2M mRNA level (Fig. 4f and g). However, at day 5, the expression of B2M started to rise again, which could explain previous findings related to differentiation into embryoid bodies.

Novel findings of downregulation of B2M during early neuroectodermal differentiation emphasize the high potential of PF14 and siRNA in studies of hES cell biology and for unraveling the regulatory networks.

## Discussion

Both RNAi and hES cells are powerful and valuable tools in basic research and biomedical applications. However, since siRNA is membrane impermeable and hES cells are refractory to commonly used transfection methods, application of RNAi in these cells has been challenging to date. Here, we showed that CPP PF14 delivers siRNA into hES cells with high efficiency without inducing side effects and thus might serve as a powerful tool in hES cell studies.

First, experiments with fluorescently labeled siRNA demonstrated high transfection efficiency of PF14 as 90% of cells were transfected already after 4 h of incubation. The slight decrease in transfected cell proportion after removal of complexes from cell medium indicates an active uptake and metabolism of siRNA [38]. Without any siRNA/PF14 particles in extracellular space to enter the cells, the fluorescent signal possibly weakened also because of cell division. Furthermore, localization of siRNA in the cytoplasm of hES cell was detected by confocal microscopy suggesting that it could induce silencing using RISC and triggering mRNA degradation as described before [39].

Next, experiments with functional siRNAs confirmed that PF14-mediated cargo delivery can be readily used to effectively knock down specific targets in hES cells. We achieved knockdown of OCT4 at least in 70% of cells at protein level and a nearly 10-fold decrease at mRNA level, which confirmed that siRNA was effectively released from endosomes and reached RISC as expected [40]. Efficient knockdown with single transfection could be partly explained by taking advantage of the passaging hESC as small cell clumps and transfection shortly after plating as previously proposed by Ma et al. and Liu et al. [16, 17]. With small clumps of cells and the addition of Rho-associated kinase (ROCK) inhibitor [41, 42], we achieved excellent viability of hESC that might be slightly affected at high siRNA/PF14 concentrations (siRNA ≥ 30 nM). Altogether, PF14 low cytotoxicity enables 24-h transfection right after plating resulting in highly efficient gene silencing even at low siRNA concentrations, which is crucial for minimizing possible sequence-related off-target effects [43, 44]. With our transfection strategy, and in contrary to report by Liu et al. [17], who reported over 90% knockdown of OCT4 at mRNA level using Lipofectamine RNAiMAX, we could omit all pre-treatment manipulations and enzymatic dissociation, both of which could influence the hES cell state. Therefore, the simplicity and compatibility with routine EDTA passaging of hES cells are essential benefits of the usage of PF14 [45].

Taking into account that downregulation of OCT4 could also be partly caused by the feedback loops altered by siRNA-induced silencing, we chose B2M as a target, which is not involved in the pluripotency network [18]. siB2M triggered modest and short-term reduction of mRNA and protein levels of B2M confirming that RNAi was indeed achieved through mRNA degradation, rather than by interfering with translation, which is the mechanism of function for miRNA (reviewed by Ipsaro and Joshua-Tor [46]). Comparison of OCT4 and B2M silencing indicates that the efficiency of knockdown depends on the target gene, as noted before [16]. However, the simplicity of the procedure opens a possibility to optimize transfection protocol for each specific target or objective of the experiment. Targetability of different genes shows that siRNA can be applied to study effects of transient or stable knockdown on the fate and functioning of hES cell. Furthermore, siRNA/PF14 complexes can silence genes of transcription factors (OCT4) and membrane proteins (B2M) and possibly many other types of effectors.

The molecular mechanisms regulating the pluripotency and differentiation of ES cells have been of great interest since their isolation in 1981 [47]. For that reason, RNAi has been widely used to dissect the roles of pluripotency factors and their targets in hES cells, but has produced contradictory conclusions [36]. For example, OCT4 has been repeatedly suggested as a factor counteracting trophectodermal differentiation [8, 30, 48]. However, more recently, it has been shown that silencing of OCT4 induces neuroectodermal rather than extraembryonal differentiation [36]. As functioning of OCT4 was discovered to be hES cell line specific and dependent on BMP4 levels, it has been suggested that discrepancies in results could be explained by the developmental state of the blastocyst from which hES cells had been derived [36]. In addition, previously published conflicting results could be attributed to growth conditions (especially growth surface and medium), as mostly mouse embryonic fibroblasts or conditioned medium, both with variable characteristics, have been used. Related to that, we used Matrigel-coated plates and chemically defined medium to assess the applicability of PF14 for studying the role of OCT4.

Regarding the functional studies, silencing of OCT4 induced morphological changes, which are specific to differentiating hES cells (i.e., larger cells, increased cytoplasmic area and flattened cells with altered shape) [3, 16, 31, 33]. Alterations at molecular level confirmed that downregulation of a single key factor of hES cells pluripotency network, OCT4, can induce differentiation towards neuroectodermal lineages. This is concordant with a previous study, which showed that downregulation of OCT4 in H9 hES cells causes a loss of pluripotency and neuroectodermal specification (characterized by decrease in *NANOG* and increase in *PAX6* expression without altering levels of *SOX2*) [36]. These findings indicate that PF14 and siRNA can be used as reliable tools for studying early embryonic development, gene function, and pluripotency network as well as directing hES cells differentiation.

Lastly, we confirmed that the method itself does not influence expression of pluripotency factors and differentiation markers. In addition, we observed a decline in B2M mRNA and protein levels upon transfection with siOCT4, which was not a side effect as estimated by silencing dynamics. Although we confirmed silencing of B2M during early neuroectodermal differentiation by experiments with neural induction medium, further analysis is required to shed light on the functional background of B2M transient silencing.

## Conclusions

In conclusion, our results show that CPP-mediated delivery of siRNA can be a powerful tool for post-transcriptional gene silencing in hES cells. The main advantages of using PF14 as siRNA delivery vector are high efficiency, low cytotoxicity, no effects on pluripotency, and easy-to-perform protocol. Knocking down expression and studying the consequences will help to clarify the role of particular gene in hES cells pluripotency and self-renewal as well as to induce differentiation towards desired cell type. All these aspects together make the presented method an essential tool for harnessing hES cells in developmental biology, regenerative medicine, and drug discovery.

## Additional files

Additional file 1: Optimization of hES cell transfection protocol. A. Representative histograms illustrating OCT4 levels in untreated, single transfected (0 h), or double transfected (0 h + 24 h; 0 h + 32 h; 0 h + 48 h) hES cells. Time points in sample name refer to the beginning of transfection. Due to low survival rates in double transfected samples, all cells were analyzed and the cell counts were compared (peak height in histogram represents cell number). siRNA was used at concentration of 30 nM. B. Numerical values illustrating OCT4 levels (OCT4-positive cells (%)) and survival rate (relative cell count) in samples presented in histograms (A). Abbreviations: UT, untreated. (EPS 224 kb)

Additional file 2: Comparison of different transfection reagents and hES cell lines. A. RT-qPCR analysis of *OCT4* mRNA level in H9 hES cells upon transfection with PF14 or Lipofectamine Stem. B. RT-qPCR analysis of *OCT4* and *B2M* mRNA levels in H1 and H9 hES cells upon treatment with respective siRNA and PF14 complexes. C. RT-qPCR analysis of representative pluripotency markers expression in H1 hES cells upon treatment with siCtrl (20 nM) and PF14 complexes. mRNA level is presented as logarithm base 2 of the fold change in gene expression between the untreated and siCtrl sample. Analyses were performed at 48 h and the data are presented as mean ± SEM (*N* = 2). All values on graphs A and B are represented relative to siCtrl sample. Abbreviations: FC, fold change; LF Stem, Lipofectamine Stem. (EPS 162 kb)

Additional file 3: Morphology of transfected and untreated cells at various time points. Representative light micrographs illustrating morphology changes at 24 h, 48 h, and 72 h induced by treatment with 20 nM siOCT4, siB2M, or siCtrl compared to untreated cells. Scale bar is 500 μm. (EPS 5008 kb)

## Abbreviations

B2M: Beta-2-microglobulin
CPP: Cell-penetrating peptide
DAPI: 4,6′-Diamino-2-phenylindole
EDTA: Ethylenediaminetetraacetic acid
hES cell: Human embryonic stem cell
iPSC: Induced pluripotent stem cell
LF Stem: Lipofectamine Stem
NIM: Neural induction medium
OCT4: Octamer-binding transcription factor 4
OTX2: Orthodenticle homeobox 2
PAX6: Paired box protein 6
pDNA: Plasmid DNA
PF14: PepFect 14
RISC: RNA-induced silencing complex
RNAi: RNA interference
ROCK: Rho-associated kinase
SCO: Splice-correcting oligonucleotide
siRNA: Small interfering RNA
SOX2: SRY (sex determining region Y) box 2

## References

[1] Thomson JA, Itskovitz-Eldor J, Shapiro SS, Waknitz MA, Swiergiel JJ, Marshall VS (1998). Embryonic stem cell lines derived from human blastocysts. Science.

[2] Nichols J, Zevnik B, Anastassiadis K, Niwa H, Klewe-Nebenius D, Chambers I (1998). Formation of pluripotent stem cells in the mammalian embryo depends on the POU transcription factor Oct4. Cell.

[3] Hyslop L, Stojkovic M, Armstrong L, Walter T, Stojkovic P, Przyborski S (2005). Downregulation of NANOG induces differentiation of human embryonic stem cells to extraembryonic lineages. Stem Cells.

[4] Chambers I, Colby D, Robertson M, Nichols J, Lee S, Tweedie S (2003). Functional expression cloning of Nanog, a pluripotency sustaining factor in embryonic stem cells. Cell.

[5] Pei D (2009). Regulation of pluripotency and reprogramming by transcription factors. J Biol Chem.

[6] Vallier L, Alexander M, Pedersen RA (2005). Activin/Nodal and FGF pathways cooperate to maintain pluripotency of human embryonic stem cells. J Cell Sci.

[7] Chen Y-G, Li Z, Wang X-F (2012). Where PI3K/Akt meets Smads: the crosstalk determines human embryonic stem cell fate. Cell.

[8] Babaie Y, Herwig R, Greber B, Brink TC, Wruck W, Groth D (2007). Analysis of Oct4-dependent transcriptional networks regulating self-renewal and pluripotency in human embryonic stem cells. Stem Cells.

[9] Boettcher M, McManus MT (2015). Choosing the right tool for the job: RNAi, TALEN, or CRISPR. Mol Cell.

[10] Fire A, Xu S, Montgomery MK, Kostas SA, Driver SE, Mello CC (1998). Potent and specific genetic interference by double-stranded RNA in Caenorhabditis elegans. Nature.

[11] McManus MT, Sharp PA (2002). Gene silencing in mammals by small interfering RNAs. Nat Rev Genet.

[12] Elbashir SM, Harborth J, Lendeckel W, Yalcin A, Weber K, Tuschl T (2001). Duplexes of 21-nucleotide RNAs mediate RNA interference in cultured mammalian cells. Nature.

[13] Luo C, Lü D, Pan J, Long M (2016). Improving the gene transfection in human embryonic stem cells: balancing with cytotoxicity and pluripotent maintenance. ACS Appl Mater Interfaces.

[14] Kim TK, Eberwine JH (2010). Mammalian cell transfection: the present and the future. Anal Bioanal Chem.

[15] Zhao M, Yang H, Jiang X, Zhou W, Bin A, Ae Z, et al. Lipofectamine RNAiMAX: an efficient siRNA transfection reagent in human embryonic. Stem Cells. 2008;40:19–26. 10.1007/s12033-008-9043-x 18327560

[16] Ma Y, Jin J, Dong C, Cheng E-C, Lin H, Huang Y (2010). High-efficiency siRNA-based gene knockdown in human embryonic stem cells. RNA.

[17] Liu H, Ren C, Zhu B, Wang L, Liu W, Shi J, et al. High-efficient transfection of human embryonic stem cells by single cell-plating and starvation. Stem Cells Dev. 2016;25:477–91. 10.1089/scd.2015.0301 26772602

[18] Hay DC, Sutherland L, Clark J, Burdon T (2004). Oct-4 knockdown induces similar patterns of endoderm and trophoblast differentiation markers in human and mouse embryonic stem cells. Stem Cells.

[19] Futaki S, Ohashi W, Suzuki T, Niwa M, Tanaka S, Ueda K, et al. Stearylated arginine-rich peptides: a new class of transfection systems. Bioconjug Chem. 2001;12:1005–11. 10.1021/bc015508l 11716693

[20] Lundin P, Johansson H, Guterstam P, Holm T, Hansen M, Langel Ü (2008). Distinct uptake routes of cell-penetrating peptide conjugates. Bioconjug Chem.

[21] Ezzat K, EL Andaloussi S, Zaghloul EM, Lehto T, Lindberg S, Moreno PMD (2011). PepFect 14, a novel cell-penetrating peptide for oligonucleotide delivery in solution and as solid formulation. Nucleic Acids Res.

[22] Veiman K-L, Mäger I, Ezzat K, Margus H, Lehto T, Langel K, et al. PepFect14 peptide vector for efficient gene delivery in cell cultures. Mol Pharm. 2013;10:199–210. 10.1021/mp3003557 23186360

[23] Margus H, Arukuusk P, Langel Ü, Pooga M (2016). Characteristics of cell-penetrating peptide/nucleic acid nanoparticles. Mol Pharm.

[24] Kaitsuka T, Tomizawa K (2015). Cell-penetrating peptide as a means of directing the differentiation of induced-pluripotent stem cells. Int J Mol Sci.

[25] Livak KJ, Schmittgen TD (2001). Analysis of relative gene expression data using real- time quantitative PCR and the 2^−ΔΔC^T method. Methods.

[26] Richard JP, Melikov K, Vives E, Ramos C, Verbeure B, Gait MJ (2003). Cell-penetrating peptides. A reevaluation of the mechanism of cellular uptake. J Biol Chem.

[27] Urgard E, Lorents A, Klaas M, Padari K, Viil J, Runnel T (2016). Pre-administration of PepFect6-microRNA-146a nanocomplexes inhibits inflammatory responses in keratinocytes and in a mouse model of irritant contact dermatitis. J Control Release.

[28] Zamore PD, Tuschl T, Sharp PA, Bartel DP (2000). RNAi: double-stranded RNA directs the ATP-dependent cleavage of mRNA at 21 to 23 nucleotide intervals. Cell.

[29] Wang D, Quan Y, Yan Q, Morales JE, Wetsel RA (2015). Targeted disruption of the β2-microglobulin gene minimizes the immunogenicity of human embryonic stem cells. Stem Cells Transl Med.

[30] Matin MM, Walsh JR, Gokhale PJ, Draper JS, Bahrami AR, Morton I (2004). Specific knockdown of Oct4 and beta2-microglobulin expression by RNA interference in human embryonic stem cells and embryonic carcinoma cells. Stem Cells.

[31] Zafarana G, Avery SR, Avery K, Moore HD, Andrews PW. Specific knockdown of OCT4 in human embryonic stem cells by inducible short hairpin RNA interference. Stem Cells. 2009;27:776–82. 10.1002/stem.5 PMC2847189 19350677

[32] Hohenstein KA, Pyle AD, Chern JY, Lock LF, Donovan PJ (2008). Nucleofection mediates high-efficiency stable gene knockdown and transgene expression in human embryonic stem cells. Stem Cells.

[33] Zaehres H, Lensch MW, Daheron L, Stewart SA, Itskovitz-Eldor J, Daley GQ (2005). High-efficiency RNA interference in human embryonic stem cells. Stem Cells.

[34] Boyer LA, Lee TI, Cole MF, Johnstone SE, Levine SS, Zucker JP (2005). Core transcriptional regulatory circuitry in human embryonic stem cells. Cell.

[35] Wang J, Rao S, Chu J, Shen X, Levasseur DN, Theunissen TW (2006). A protein interaction network for pluripotency of embryonic stem cells. Nature.

[36] Wang Z, Oron E, Nelson B, Razis S, Ivanova N (2012). Distinct lineage specification roles for NANOG, OCT4, and SOX2 in human embryonic stem cells. Cell Stem Cell.

[37] Drukker M, Katz G, Urbach A, Schuldiner M, Markel G, Itskovitz-Eldor J (2002). Characterization of the expression of MHC proteins in human embryonic stem cells. Proc Natl Acad Sci U S A.

[38] Hällbrink M, Oehlke J, Papsdorf G, Bienert M (2004). Uptake of cell-penetrating peptides is dependent on peptide-to-cell ratio rather than on peptide concentration. Biochim Biophys Acta Biomembr.

[39] Hannon GJ (2002). RNA interference. Nature.

[40] Wilson RC, Doudna JA (2013). Molecular mechanisms of RNA interference. Annu Rev Biophys.

[41] Watanabe K, Ueno M, Kamiya D, Nishiyama A, Matsumura M, Wataya T (2007). A ROCK inhibitor permits survival of dissociated human embryonic stem cells. Nat Biotechnol.

[42] Claassen DA, Desler MM, Rizzino A (2009). ROCK inhibition enhances the recovery and growth of cryopreserved human embryonic stem cells and human induced pluripotent stem cells. Mol Reprod Dev.

[43] Birmingham A, Anderson EM, Reynolds A, Ilsley-Tyree D, Leake D, Fedorov Y (2006). 3′ UTR seed matches, but not overall identity, are associated with RNAi off-targets. Nat Methods.

[44] Caffrey DR, Zhao J, Song Z, Schaffer ME, Haney SA, Subramanian RR (2011). siRNA off-target effects can be reduced at concentrations that match their individual potency. PLoS One.

[45] Beers J, Gulbranson DR, George N, Siniscalchi LI, Jones J, Thomson JA (2012). Passaging and colony expansion of human pluripotent stem cells by enzyme-free dissociation in chemically defined culture conditions. Nat Protoc.

[46] Ipsaro JJ, Joshua-Tor L (2015). From guide to target: molecular insights into eukaryotic RNA-interference machinery. Nat Struct Mol Biol.

[47] Evans MJ, Kaufman MH (1981). Establishment in culture of pluripotential cells from mouse embryos. Nature.

[48] Fong H, Hohenstein KA, Donovan PJ (2008). Regulation of self-renewal and pluripotency by Sox2 in human embryonic stem cells. Stem Cells.

